# Tafamidis for cardiac transthyretin amyloidosis: application in a real-world setting in Germany

**DOI:** 10.1007/s00392-023-02163-x

**Published:** 2023-02-23

**Authors:** Caroline Morbach, Aikaterini Papagianni, Sandra Ihne-Schubert, Vladimir Cejka, Maximilian Steinhardt, Georg Fette, Melissa Held, Andreas Geier, Hermann Einsele, Stefan Frantz, Stefan Knop, Claudia Sommer, Stefan Störk

**Affiliations:** 1 Department Clinical Research and Epidemiology, Comprehensive Heart Failure Center, University Hospital Würzburg, Am Schwarzenberg 15, 97078 Würzburg, Germany; 2 Department Medicine I, University Hospital Würzburg, Würzburg, Germany; 3 Department Neurology, University Hospital Würzburg, Würzburg, Germany; 4 Department Medicine II, University Hospital Würzburg, Würzburg, Germany; 5 Department of Haematooncology, Clinic for Internal Medicine, Diabetology, Gastroenterology, Tumour Medicine and Palliative Medicine, Medius Klinik Nürtingen, Nürtingen, Germany; 6 Service Center Medical Informatics, University Hospital Würzburg, Würzburg, Germany; 7 Department Internal Medicine 5, Hemato-Oncology, Paracelsus Medical University, Nuremberg, Germany

Sirs,

Based on the results of the Tafamidis Treatment for Patients with Transthyretin Amyloid Cardiomyopathy trial (ATTRACT) [1], tafamidis (61 mg once daily) was approved in Germany in February 2020 for the treatment of cardiac transthyretin amyloidosis. According to the *Fachinformation* [2], tafamidis 61 mg can be prescribed, in Germany, to any patient with a diagnosis of cardiac transthyretin amyloidosis (ATTR) confirmed by a physician experienced in the treatment of amyloidosis or cardiomyopathy. It is recommended to initiate treatment “as early as possible in the course of the disease” acknowledging that “evidence is less strong in patients with more advanced heart failure”. Further, tafamidis is recommended to be used “with caution in patients with impaired renal or hepatic function”.

We here report the prescription of tafamidis by the Interdisciplinary Amyloidosis Center Northern Bavaria, a tertiary care center in southern Germany. Data were derived from electronic sources of the hospital information system via its proprietary data ware house [3] (Ethics Committee waiver #2021/1011/01). At the Amyloidosis Center, according to internal standard procedures [4], diagnosis of cardiac transthyretin amyloidosis is confirmed and future therapy is determined [5] by an interdisciplinary board of cardiologists, neurologists, and hematologists. Every 6 months, patients undergo repeat clinical evaluation including echocardiography and assessment of renal, hepatic, and cardiac biomarkers, and respective clinical results as well as potential adaptations in the therapeutic strategy are discussed in the board.

In ATTRACT, tafamidis therapy resulted in a reduction of all-cause mortality risk with the Kaplan–Meier curves diverging after approximately 18 months [1]. Hence, a benefit of tafamidis therapy can be assumed in patients with a life expectancy > 1.5 to 2 years. At our center, the recommendation for an individual patient to receive tafamidis therapy is based on the above cited *Fachinformation *[2], the inclusion and exclusion criteria of ATTRACT [1], as well as on the patient´s overall clinical presentation in the absence of severe life-limiting comorbidities.

Between January 2020 and January 2022, 144 patients with confirmed diagnosis of cardiac ATTR (mean age 75 ± 11 years; *n* = 113 men [78%]) presented at our center. Of those, *n* = 107 had ATTRwt, *n* = 15 ATTRv, and *n* = 22 were without definite diagnosis due to lack of genetic testing.

Of those, 101 patients (aged 77 ± 8 years, *n* = 81 men [80%]) qualified for tafamidis therapy. Important characteristics of these patients are described in the Table 1. The majority of patients (*n* = 95) received continuous tafamidis prescription by our center, whereas, in six patients, tafamidis was initiated at the center, yet continued by a primary care physician or cardiologist.

**Table 1:** Characteristics of patients with confirmed cardiac ATTR qualifying for tafamidis therapy

	All patients N = 101	Men N = 81	Women N = 20	ATTRwt N = 85 (72 men)	ATTRv N = 5 (4 men)
Age (years)	77 (8)	77 (6)	77 (12)	77 (6)	60 (18)
NYHA functional class					
I	10 (10)	9 (11)	1 (5)	8 (9)	2 (40)
II	36 (36)	28 (35)	8 (40)	35 (41)	0 (0)
III	55 (54)	44 (54)	11 (55)	42 (49)	3 (60)
IV	0 (0)	0 (0)	0 (0)	0 (0)	0 (0)
Disease stage (according to Gillmore et al., Eur Heart J. 2018)					
I	47 (47)	39 (48)	8 (40)	41 (48)	0 (0)
II	31 (31)	25 (31)	6 (30)	26 (31)	2 (40)
III	23 (23)	17 (21)	6 (30)	18 (21)	3 (60)
Heart rate (min^−1^)	67 (59; 78)	66 (59; 77)	70 (61; 86)	66 (59; 77)	73 (67; 88)
QRS duration (ms)	126 (102; 153)	128 (105; 155)	98 (92; 105)	128 (104; 155)	95 (89; 103)
Six-minute walk distance (m)	300 (240; 390)	320 (250; 400)	232 (160; 325)	320 (240; 400)	280 (200; 600)
Biomarkers					
NT-proBNP (pg/ml)	2619 (1287; 4007)	2619 (1287; 3937)	2872 (1203; 4203)	2531 (1318; 3775)	319 (34; 2680)
High-sensitive troponin T (pg/ml)	42.9 (35.1; 68.3)	44.3 (35.2; 72.3)	37 (28; 46)	43 (35; 70)	26 (12; 43)
eGFR (ml/min/1.73 m^2^)	51 (38; 67)	50 (38; 67)	52 (37; 69)	51 (41; 67)	72 (66; 102)
Echocardiography					
Interventricular septum end-diastolic thickness (mm)	20 (16; 21)	20 (17; 21)	19 (16; 20)	20 (17; 21)	16 (12; 24)
Posterior LV wall end-diastolic thickness (mm)	15 (13; 17)	15 (13; 17)	16 (14; 18)	15 (14; 17)	13 (9; 16)
LV mass index (g/m^2^)	168 (142; 208)	173 (142; 194)	160 (129; 223)	177 (143; 223)	140 (60; 193)
LV end-diastolic volume (ml)	98 (76; 135)	105 (81; 146)	78 (57; 96)	96 (77; 139)	101 (93; 137)
LV ejection fraction (%)	54 (45; 60)	53 (45; 60)	57 (45; 63)	54 (46; 60)	58 (46; 64)
LV stroke volume (ml)	71 (58; 86)	74 (59; 88)	62 (51; 79)	74 (60; 86)	77 (63; 92)
LV global longitudinal strain (− %)	11 (9; 13)	10 (9; 12)	12 (8; 15)	11 (9; 13)	12 (8; 23)
Cardiac output (l/min)	4.7 (3.9; 5.7)	4.8 (4.1; 5.8)	4.0 (3.5; 4.8)	4.7 (3.9; 5.7)	4.9 (4.1; 7.7)
E/e′	23 (16; 28)	22 (16; 26)	26 (16; 32)	23 (17; 27)	12 (8; 23)
RV wall thickness (mm)	7 (6; 9)	7 (6; 9)	8 (6; 10)	8 (6; 9)	6 (6; 7)
TAPSE (mm)	15 (13; 19)	16 (13; 20)	15 (14; 17)	16 (12; 19)	19 (15; 23)
TR max PG (mmHg)	34 (28; 41)	33 (28; 38)	38 (31; 42)	34 (28; 41)	29 (19; 29)

Data are frequency (percent), mean (SD), or median (quartiles). Data were available in > 70% of patients

*NYHA* New York Heart Association, *NT-proBNP* N-terminal pro-brain-natriuretic peptide, *eGFR* estimated glomerular filtration rate according to CKD-EPI formula, *LV* left ventricle, *E* early mitral inflow velocity, *e′* early LV relaxation velocity, *RV* right ventricle, *TAPSE* tricuspid annulus peak systolic excursion, *TR max PG* maximal systolic pressure gradient across tricuspid valve

Patients qualifying for tafamidis therapy in clinical routine (Table 1) were slightly older and more often of female sex when compared to the ATTRACT population (there: 10% women, 74 ± 7 years) [1]. They further showed higher left ventricular (LV) wall thickness, but more favorable functional parameters when compared to ATTRACT (there: LV ejection fraction 49%, LV global longitudinal strain − 9%) [1]. Until now, the median duration of therapy of the total sample was 8.5 (quartiles 4; 18) months. In the subgroup of patients with more than 12 months of therapy (*n* = 37, aged 75 ± 7 years, 84% men), in the time period between first prescription of tafamidis to the hitherto last presentation at our center, we observed the following changes—median (Q1; Q3) change: NT-proBNP + 4 (− 548; 788) pg/mL, estimated glomerular filtration rate + 5 (− 1; + 12) ml/min, high-sensitive troponin + 2.4 (− 5.0; 8.5) pg/ml, LV ejection fraction + 1 (− 6; 6) %, interventricular septal wall thickness of + 2 (− 1; 7) mm, E/e′ + 0 (− 4; 5), tricuspid annular plane systolic excursion of − 1 (− 3; 2) mm. This is in line with recent publications where detailed clinical phenotyping and serial cardiac magnet resonance imaging revealed that tafamidis did not improve the patients´ cardiac phenotype, but slowed down disease progression [6, 7].

Up to now, tafamidis therapy was terminated after thorough discussion in the interdisciplinary board in five patients due to newly developed malignancy (*n* = 2), end-stage renal disease (*n* = 1), and terminal heart failure due to advanced cardiac amyloidosis (*n* = 2).

Tafamidis therapy was well tolerated in almost all patients (termination due to intolerance: *n* = 1). Careful monitoring of the potential long-term benefits in patients treated in routine care is required. Regarding the associated costs of tafamidis, there is an urgent need to internationally agree on criteria guiding both initiation and also termination of tafamidis therapy.

## Data Availability

Data will be made available upon request.
